# Exploring the Preferences and Behavioral Trends of e-Patients in Psychosomatics Towards Telemedicine During and Post COVID-19 Pandemic: Cross-Sectional Analysis

**DOI:** 10.2196/74167

**Published:** 2026-01-16

**Authors:** Moritz Mahling, Alexander McQueeney, Teresa Festl-Wietek, Ken Masters, Stephan Zipfel, Anne Herrmann-Werner, Caroline Rometsch

**Affiliations:** 1 Deanery of Students’ Affairs, University of Tübingen, Tübingen, Germany; 2 Department of Diabetology, Endocrinology, Nephrology, Section of Nephrology and Hypertension, University Hospital Tübingen, Tübingen, Germany; 3 Tübingen Institute for Medical Education (TIME), Faculty of Medicine, Tübingen, Germany; 4 Medical Education and Informatics Department, College of Medicine and Health Sciences, Sultan Qaboos University, Muscat, Sultanate of Oman; 5 Department of Psychosomatic Medicine and Psychotherapy, University Hospital Tübingen, Osianderstr. 5, Tübingen, 72074, Germany, 49 7071 29-86719; 6 Department of Psychosomatic Medicine and Psychotherapy, Otto-von-Guericke University of Magdeburg, Magdeburg, Germany

**Keywords:** COVID-19, pandemic, eHealth, e-patient, preferences, gender differences

## Abstract

**Background:**

COVID-19 accelerated the adoption of health services, with a growing number of psychosomatic patients turning to the internet for health-related decisions. This study explored changes in communication behavior, information-seeking habits, and post-pandemic consultation preferences among psychosomatic patients during and after COVID-19.

**Objective:**

This study explored changes in communication behavior, information-seeking habits, and post-pandemic consultation preferences among psychosomatic patients during and after COVID-19.

**Methods:**

In a cross-sectional study, 150 adult patients (>18 y) from the psychosomatic outpatient department in Tübingen, Germany, were invited to complete an ad hoc questionnaire to identify e-patients’ preferences related to communication, information-seeking behavior, subjective explanations, and postpandemic preferences. Group comparisons and multiple linear regression analyses were conducted.

**Results:**

The study revealed a slight increase in online-based communication between patients and caregivers (eg, caregivers’ system use +10.8%; live video consultations +30.8%), as well as in patient–patient interactions (eg, online correspondence +16.9%). Significant group differences were observed for social media correspondence by patient age (*χ*^2^¬=17.44, *P*<.001) and for live video consultations by gender (*χ*^2^¬=70.17, *P*<.001). For both age and gender, significant group differences were found in the use of medical videos (age *χ*^2^¬=6.36, *P*=.04; gender *χ*^2^¬=76.70, *P*<.001). Age, gender, and preferences for live video consultations were identified as significant predictors (*F*_1,11_=14.195, *P*<.001, *R*^²^=0.299) of patients’ future preferences for on-site versus online consultations.

**Conclusions:**

Although a slight increase in online-based communication was observed, resilience in patient–caregiver and patient–patient communication indicated relative stability under challenging circumstances. Older adults and female patients expressed a preference for on-site consultations, emphasizing the significance of in-person care. Male patients demonstrated greater openness towards online consultations, indicating potential for expansion of eHealth services. Identifying preferences is a core essential for providing future eHealth implementations that account for diverse patient needs and for designing care offerings. Incorporating these findings may enhance patient engagement and satisfaction in the evolving landscape of eHealth services for better health care outcomes.

## Introduction

eHealth refers to the delivery of health services and information via the internet or other electronic technologies [1]. During the COVID-19 pandemic, the role of eHealth in medicine achieved its highest level of importance while combating the deadly virus [2]. The accompanying social distancing norms further expedited digital developments in this field [3]. eHealth has presented promising evidence for its effectiveness [4,5], improving care quality, while reducing health care costs [6]. During the pandemic, eHealth services facilitated communication between caregivers and patients while maintaining distancing policies, thereby reducing hospital visits and direct contact [3,7,8]. During the COVID-19 pandemic, patient–caregiver communication was conducted via webcam-enabled computers, smartphones, and telephone conferencing [9,10]. This assisted caregivers in making patient-centered clinical decisions [11].

Reflecting the growing emphasis on patient involvement in decision-making, eHealth has enabled patients to use the internet as a source of empowerment. Consequently, patients are able to play a more active role in their own medical care, for example, by presenting information during consultations [12] and making informed decisions [13]. Patients who use the internet to inform themselves about health are termed “e-patients” [14,15]. In this study, e-patients refer to patients who actively use digital technologies to seek health information, which distinguishes them from broader digital health users who may passively use online health services without engaging in informed decision-making.

As nonmedical experts, e-patients often seek practical advice on social media [16,17]. The use of these platforms is influenced by patients’ awareness of health problems and their attempts to reduce the risk of illness [3]. Apart from social media, e-patients use general search engines (eg, Google), medical sites (eg, UpToDate, WebMD), and medical journals (eg, BMJ, Lancet) for information [18-20]. Moreover, health apps and wearables provide relevant health-related information (eg, tracking COVID-19 infection) that has been incorporated into medical treatments [21]. This shift has resulted in a more informed e-patient population than in previous decades, as is evident in patient–physician interactions [22].

The growing literature on patients’ experiences with eHealth during COVID-19 indicates high satisfaction rates, including such factors as dealing with concerns, communication, usefulness, reliability, time savings, access, and cost-efficiency [23]. In managed care, eHealth is pivotal in promoting patient-focused care. However, challenges remain [24], particularly the technical demands of using eHealth [25]. To ensure that patient involvement remains a core element in the future implementation of eHealth after the COVID-19 pandemic [26], further investigation into patients’ preferences and behaviors is required.

Thus, this study aims to examine patient adoption of eHealth, changes in patients’ behavior, and patients’ explanations for those changes during and after the COVID-19 pandemic. Specifically, it focuses on the dimensions of preferences and experiences in communication, information acquisition and the use of health apps and wearables in a German psychosomatic outpatient clinic.

## Methods

### Study Design

This cross-sectional study was conducted from October 1, 2020, to March 31, 2021, at the Outpatient Department of Psychosomatic Medicine and Psychotherapy in Tübingen, Germany. A total of 175 patients aged 18 years and older were invited to voluntary participate and were asked to complete a specially-designed questionnaire. It took approximately 15 to 20 minutes for patients to complete the written questionnaire.

Patients were seen by a medical doctor or psychologist in the outpatient department for diagnostic consultations, including clinical interviews and psychometric assessments. Admission to the outpatient service was voluntary, and most patients had been referred by general practitioners, psychiatrists, or psychologists because of mental health complaints. Participation in the study was entirely voluntary. Patients with insufficient German language proficiency or severe psychiatric disorders were excluded, and no responses were excluded during data analysis.

### Ethical Considerations

This study was conducted in accordance with the ethical standards of the Declaration of Helsinki [27], and was approved by the Ethics Committee of Tübingen Medical Faculty (no. 243/2022BO2). Patients provided written, informed consent prior to participation, after being informed about the study’s aim, the voluntary nature of participation, their right to withdraw without consequences, and the measures taken to ensure data security. For analysis, data were anonymized to ensure that patients could not be identified. The authors did not have access to information that could identify individual participants during or after data collection. No incentives were provided. No images of individual participants or users are included in the manuscript or supplementary material.

### Assessment Instruments

Drawing from the literature on current e-patient activities and changes in physician–patient interactions during the COVID-19 pandemic [28,29], an ad hoc questionnaire was created to examine e-patients’ activities and the impact that COVID-19 had on them. The think-aloud technique was used in the development of the ad hoc questionnaire items, as no validated questionnaire on e-patients’ activities existed [30]. The questionnaire assessed four main domains: (1) changes in communication compared to 2019, (2) changes in patients’ use of eHealth compared to 2019, (3) patients’ interpretations of changes attributable to COVID-19, and (4) patients’ preferences post-COVID-19 pandemic. Items regarding communication and changes in patients’ use of eHealth explored whether patients’ health-related electronic activity had changed (eg, decreased, increased, no change) since December 2019 in terms of communication with caregivers or other patients, specifying the medium used (eg, video calls, emails, medical websites, wearable devices, apps) or the method of information acquisition (eg, medical books, videos, medical journals, networking). Changes were quantified on a 3-point Likert scale (1=‘not at all’, 2=‘somewhat’, 3=‘very’) to evaluate subjective explanations linked to COVID-19. Patients’ preferences for future interactions (face-to-face vs eHealth) were assessed using their reaction to the following statement: “After things return to normal, I would like to interact with my doctors in the following way: only face-to-face; mostly face-to-face; face-to-face and online equally; mostly online; or only online.”

### Statistical Analysis

Statistical analyses were run using SPSS software (version 28; IBM Corp) [31]. Sociodemographic data and responses to the ad hoc questionnaire were evaluated descriptively. Respondents were categorized into two age groups based on the mean age (<37 y and ≥37 y). Group differences were determined using *χ*^2^ tests. The statistically significance was set at *P*<.05. A multiple linear regression analysis was performed with the consultation format preference (on-site vs online) as the dependent variable, assessed on a 5-point ordinal scale (only face-to-face, mostly face-to-face, face-to-face and online equally, mostly online, and only online). For analysis, this variable was treated as continuous to approximate the linear trend across levels, enabling the use of multiple linear regression. This approach was chosen because the preference scale reflects a spectrum of consultation preferences rather than discrete categories. The potential predictors incorporated in the model included age, gender, communication preferences, and modes of medication and information acquisition. These predictors were selected based on their theoretical relevance from prior studies [25,32]. Multicollinearity was assessed using variance inflation factors (VIF), and no issues were identified (all VIF <2). Model fit was evaluated using *R*² and the Durbin–Watson statistic to prove autocorrelation in residuals.

## Results

### Sociodemographic Data

A total of 150 patients participated in the study, yielding a response rate of 85.7%. The mean age of the participants was 37.06 (SD 12.4) years, with the majority being female (n=88, 58.7%; unspecified n=41, 27.3%). Most patients (92/125, 73.6%) reported undergoing an in-person examination in the department, while 33/150 patients (22.0%) reported an online consultation. In-person examinations were cited as the preferred consultation type by 70.9% of patients (61/86), with no significant differences observed regarding age or gender.

### Changes in Patients’ Communication in Relation to the Timeframe of 2019

The results indicate an increase in online-based communication methods since 2019, including both written communication (eg, email, social media) and direct consultations (eg, via electronic systems, video calls). However, there was a noted decrease in live video consultations among 51/133 patients (38.3%). Patient-to-patient communication did not show significant changes. Significant group differences were observed for social media correspondence by age (*χ*^2^=17.44, *P*<.001). Regarding gender, significant group differences were found for live video consultations (*χ*^2^=70.17, *P*<.001) (see Table 1).

**Table 1.** Changes in communication behaviors among patients with mental health complaints attending the outpatient department of psychosomatic medicine and psychotherapy at Tübingen University Hospital, Germany (cross-sectional study, October 2020 to March 2021), compared with the prepandemic year 2019.

Communication partner, eHealth type, and changes in use	Total responses, n (%)
Correspondence with physicians	
Changes in email correspondence (n=90)	
Indifferent	51 (56.7)
Increase	37 (41.1)
Decrease	2 (2.2)
Social media correspondence (n=83)	
Indifferent	71 (85.5)
Increase	5 (6.0)
Decrease	7 (8.4)
Patient–physician communication	
Caregivers’ system (n=102)	
Indifferent	67 (65.7)
Increase	11 (10.8)
Decrease	24 (23.5)
Live video consultation (n=133)	
Indifferent	41 (30.8)
Increase	41 (30.8)
Decrease	51 (38.3)
Patient–patient correspondence	
Internet use (n=83)	
Indifferent	60 (72.3)
Increase	14 (16.9)
Decrease	9 (10.8)

### Changes in Patients’ E-Health Use in Relation to the Timeframe of 2019

Aligned with the results on communication, an increase was observed for wearables and eHealth apps. However, no significant changes were observed regarding the purchase of medication and obtaining medical information via the internet. However, when closely examining the increase in obtaining online medical information, the greatest growth is observed for online medical books and videos compared to the others.

Significant group differences were detected concerning online purchase of medication (*χ*^2^=6.94, *P*=.03), medical books (*χ*^2^=9.43, *P*=.009), medical videos (*χ*^2^=6.36, *P*=.04), medical journals (*χ*^2^=9.53, *P*<.05), and wearables (*χ*^2^=8.92, *P*<.05), in regard to the patient’s age. Regarding gender, significant group differences were found for medical videos (*χ*^2^=76.70, *P*<.001), medical websites (*χ*^2^=43.67, *P*<.001) (see Table 2).

**Table 2.** Changes in behavior regarding e-health activities by patients with mental health complaints attending the outpatient Department of Psychosomatic Medicine and Psychotherapy at Tübingen University Hospital, Germany (October 2020 to March 2021), compared to the pre-pandemic year 2019).

Category, specification, and changes in use	Total , n (%)
Medication	
Online purchase (n=87)	
Indifferent	54 (62.1)
Increase	30 (20)
Decrease	3 (3.4)
Obtaining medical information	
Medical books (n=87)	
Indifferent	62 (71.3)
Increase	20 (23.0)
Decrease	5 (5.7)
Medical videos (n=132)	
Indifferent	58 (43.9)
Increase	27 (18.0)
Decrease	47 (35.6)
Medical websites (n=107)	
Indifferent	61 (57.0)
Increase	22 (20.6)
Decrease	24 (22.4)
Medical journals (n=87)	
Indifferent	71 (81.6)
Increase	7 (8.0)
Decrease	9 (6.0)
Networking sites (n=88)	
Indifferent	65 (73.9)
Increase	13 (14.8)
Decrease	10 (11.4)
eHealth technology	
Wearables (n=133)	
Indifferent	100 (75.2)
Increase	21 (15.8)
Decrease	12 (9.0)
Health applications (n=104)	
Indifferent	53 (51.0)
Increase	46 (44.2)
Decrease	5 (3.3)

### Changes Due to COVID-19

**Figure 1.**
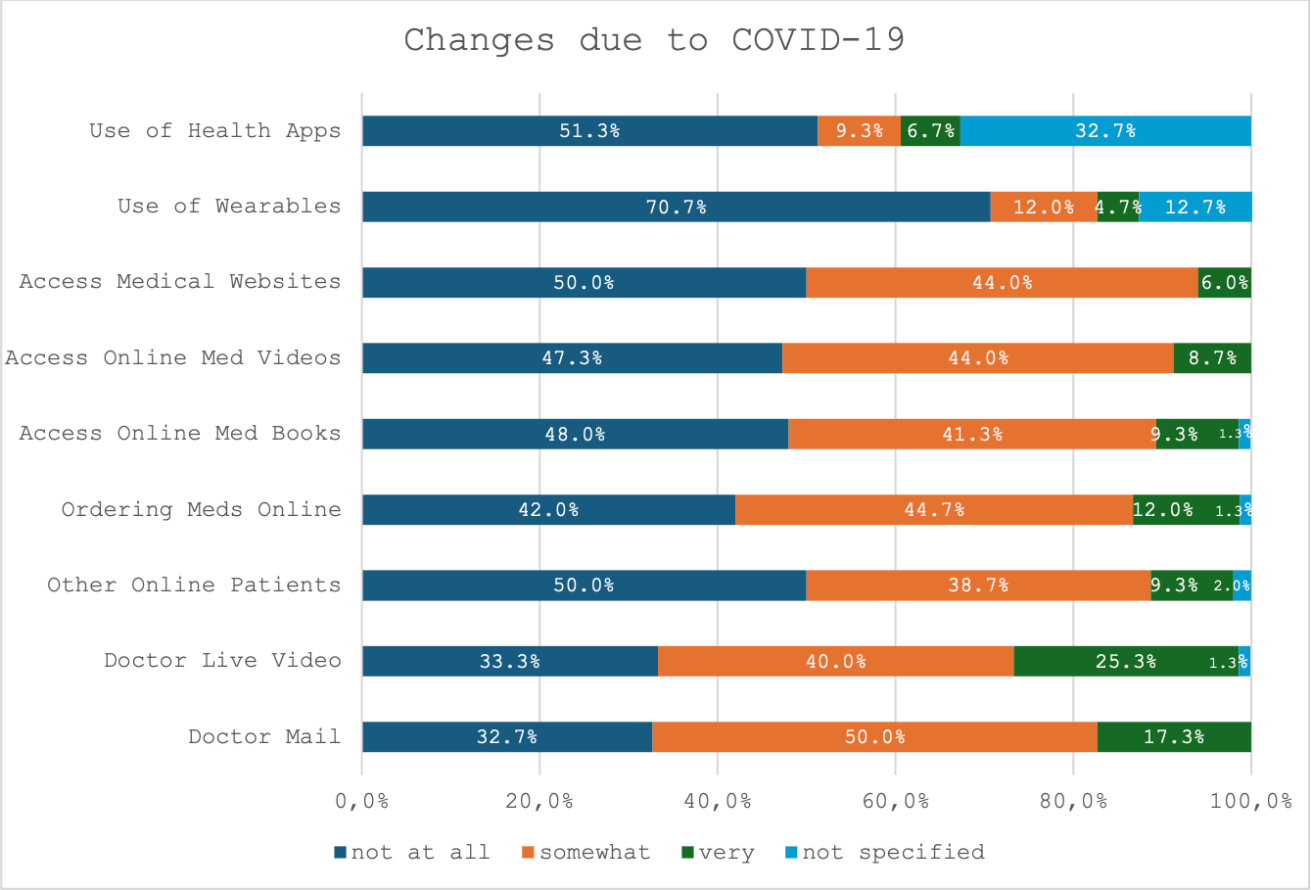
Changes in patient communication, information acquisition, online purchase of medication, and wearables and eHealth apps among patients attending the outpatient department of psychosomatic medicine and psychotherapy at Tübingen University Hospital, Germany (cross-sectional study, October 2020 to March 2021) caused by the COVID-19 pandemic.

Patients’ communication was moderately affected by the COVID-19 pandemic, with a mean score of 1.69 (SD 0.53). A similar impact was observed in their search for online information (mean 1.24, SD 0.47). However, a more significant change due to the pandemic was observed in two aspects: the online acquisition of medication (mean 1.70, SD 0.68) and the use of eHealth technology (mean 2.23, SD 0.45). For a detailed analysis of the changes caused by COVID-19, see Figure 1.

### Explanation of Changes Due to COVID-19

In terms of patient communication, changes in email correspondence were attributed to COVID-19 by 16/90 patients. Social media correspondence was reported to have changed due to COVID-19 by 3/83 patients. A total of 8/102 indicated that the caregivers’ system had changed, while 27/102 patients reported alterations in live video consultations.

With respect to eHealth use, the online purchase of medication was identified as having changed due to COVID-19 by 7/87 patients. A total of 5/87 patients reported informing themselves more via medical books. Changes in the use of medical videos were reported by 4/132 patients , while 3/107 patients reported changes in the use of medical websites. A total of 3/87 patients indicated changes in the use of medical journals. Lastly, the use of wearable technology was reported to have subjectively increased due to COVID-19 by 5/130 patients.

### Patients’ Post-COVID-19 Pandemic Preferences

After most restrictions due to COVID-19 were lifted, most patients (n=120/147, 81.7%) stated a preference for seeing a medical caregiver on-site. Correspondingly, patients preferred patient–patient interactions to be on-site (n=88/145, 60.7%). Approximately, one-third of patients (n=48/145, 32.0%) were open to ordering medication both on-site and online, although most patients (n=81/145, 55.1%) chose the on-site option. Finally, when obtaining medical information, patients regarded on-site and online channels as equal (n=66/146, 45.2%).

### Predictors of Preference for on-Site vs Online Consultations

The results of the multiple linear regression analysis revealed a statistically significant model (*F*_1,11_=14.195, *P*<.001) that explained patients’ future preferences regarding on-site versus online consultations. Significant predictors included age (B=−0.011, SE=0.005, *t*=−2.29; *P*=.02), gender (B=0.491, SE=0.113, *t*=4.35; *P*<.001), and changes in live video consultation use during COVID-19 (B=0.270, SE=0.084, *t*=3.20; *P*=.002). This finding suggests that patients of older age are more inclined towards on-site consultations. Furthermore, the data indicates that males prefer online consultations, while female patients show a tendency towards on-site consultations. An increase in the preference for live video consultations due to COVID-19 was positively correlated with a willingness to engage in online consultations. However, online communication via email (*P*=.95), online searches for medical information (books (*P*=.70) or videos (*P*=.12)) and online medication purchases (*P*=.11) were not found to be statistically significant predictors (see Table 3).

**Table 3.** Multiple linear regression analysis identifying predictors of patients’ preferences for on-site versus online consultations in a cross-sectional study of patients with mental health complaints at the outpatient department of psychosomatic medicine and psychotherapy, Tübingen University Hospital, Germany (October 2020–March 2021).

Variables	*R* ^²^ ^a^	SE^b^	B^c^	t test^d^	95% CI	*P* value
Constant	1781	0.344	—	5181	1101 to 2461	<,001^e^
Age	−.011	0.005	−0.169	−2289	−0.020 to −0.001	.02
Gender	0.491	0.113	0.389	4347	0.268 to 0.715	<.001^e^
Online purchase of medication	−0.159	0.099	−0.136	−1599	–0.356 to –0.038	.11
Correspondence via mail	−0.006	0.094	−,006	−0.069	–0.192 to 0.179	.95
Live-video consultations	0.270	0.084	0.262	3204	0.104 to 0.437	.002^f^
Information via medical books	0.059	0.151	0.048	0.391	−0.240 to 0.358	.67
Information via medical videos	−0.245	0.156	−0.196	−1573	–0.553 to 0.063	.12

a *R*²: coefficient of determination.

b SE: standard error.

c B: unstandardized regression coefficient.

d T: t statistic.

e *P*<.001.

f *P*<.05.

The model demonstrates a sufficient fit with an *R*² of 0.299, indicating that 29.9% of the variation can be explained by the independent variables. The Durbin–Watson test statistic 1.354, suggests no evidence of autocorrelation in the residuals. Additionally, the collinearity diagnostics did not indicate any multicollinearity.

## Discussion

This study explored alterations in eHealth patients’ behavior and their inclinations towards eHealth throughout and following the COVID-19 pandemic. This investigation demonstrated slight variations in how patients and caregivers communicated, as well as the interactions between patients themselves. Two notable shifts due to the pandemic were evident: an increase in online obtaining of medication and an increasing deployment of eHealth technology. This study determined that age and gender were key factors influencing patients’ future decisions for in-person or virtual consultations. Older adults and female patients leaned towards on-site consultations, while male patients preferred online consultations. Furthermore, a preference for live video consultations predicted patients’ openness towards online consultations.

 eHealth reduces disease transmission and is especially suitable for self-isolating or high-risk patients [33]. Its growth, accelerated not only by the COVID-19 pandemic, presents several advantages, such as protection, time-saving, and cost reduction, yet it also carries disadvantages, such as difficulties in building a stable relationship between caregivers and patients, assessment challenges, technological issues, and data protection concerns [34,35].

This study’s findings underlined, on the one hand, a significant increase in using online-based communication but also showed no remarkable changes in its overall utilization. This resilience of communication indicated that patients made only minimal changes to their communication behavior even though social distancing was strongly regulated by the German government. This is in line with international discrete choice experiments indicating a top preference for in-person consultations for most clinical scenarios [36,37]. To sustain the eHealth revolution in health care delivery [38], an in-depth understanding of communication, currently lacking, is necessary.

The research identified crucial points for a successful implementation of eHealth. First, a clear differentiation in e-health applications is needed, as some authors suggest (eg, specific diseases, specialties, technologies, clinical problems [39]). Second, comprehensive investigations must be conducted to provide evidence (eg, chronic pain [40], oncology [41], palliative care [42]). Third, eHealth communication necessitates best practices (eg, use of plain language, reducing information overload, application of teach-back [43]), ideally provided by national or international organizations such as the World Health Organization [44].

 While adhering to best communication practices, patients’ information level must be respected. Reasons for information-seeking vary widely, from discussing specific diseases to social and emotional support through peer-to-peer interactions [45]. The increasing health-related internet use by patients results in both positive and negative effects on the caregiver–patient relationship [46]. Positive effects were observed when patients openly discussed their findings with caregivers, thereby enabling active participation in decision-making, which alters traditional consultation models [47].

A primary objective of eHealth may also be a central aspect in guiding patients within the framework of managed care programs [48]. Conversely, misleading health information may have a serious impact on an individual’s health or mental state [49], such as the phenomenon of cyberchondria [50], which involves excessive fear of symptoms resulting from reviewing of search results and references on the Web, higher medical costs [51], and overall lower quality of life due to poor health care management [52]. This study’s analysis showed only a slight increase in online-based information-seeking; however, during the pandemic, most studies found an increased desire to understand the virus’s risk situation and to cope with uncertainty [53].

 Wearables and eHealth apps played a vital role in detecting the spread of the coronavirus [54], and their usage for monitoring physical activity has risen [55]. This aligns with an increase in the use of wearables and e-health apps in this sample. Wearables and health-related apps are promising tools for managing long COVID [56] and, in general, for empowering patients to take responsibility for their health and care [57,58]. In research, wearables have become essential for data collection in population-based research, experimental outcome assessment, prognostic forecasting, and explorative analysis of big data sets [59].

In addition to the rise of wearables, online medication procurement also increased, in line with recent research [60]. However, a large number of online pharmacies have failed to comply with regulations [61], leading to deaths [62] and emphasizing the need for greater awareness and stricter government laws [63].

 Despite substantial literature demonstrating high satisfaction with eHealth across all ages and genders [23,64], this investigation revealed a clear preference for on-site consultations, in line with recent findings [32]. This may be specific to the psychosomatic sector, where comprehensive examinations of both psyche and body are part of daily practice. To elaborate on these findings, regression analysis provided more insights: older adults and female patients expressed a preference for on-site consultations, highlighting the importance of personalized, in-person care. This aligns with findings from large and international surveys that especially older people prefer in-person consultations [65] and also supports the argument that e-health may not completely replace face-to-face consultations [38]. Conversely, male patients demonstrated greater openness towards online consultations. Recent studies have found a correlation between male sex and satisfaction with treatment plan or follow-up via eHealth [66], controversially also female sex was associated with higher satisfaction of eHealth services [67]. Further influencing factors, such as positive promotion of e-health by caregivers [68], as well as personality structure or cultural background, need to be further explored.

 Limitations of this study include its cross-sectional design, which captures a retrospective snapshot of patient behavior without enabling the tracking of changes over time. Moreover, while the ad hoc questionnaire addressed context-specific topics, it was not formally validated, which restricts the reliability and generalizability of its measures. Although the think-aloud method was used during development to improve clarity and content relevance, no psychometric evaluation was conducted prior to its use. This limitation may limit the generalizability and interpretability of the findings. However, evidence suggests that data from ad hoc clinician-generated patient questionnaires can be aggregated into valid factors [69] which indicates ad hoc questionnaires may still provide meaningful insights into patient behaviors and preferences.

Potential selection bias due to the voluntary participation of patients may also be a limitation. An additional limitation is missing data regarding the participants’ gender, which may have introduced bias or reduced the validity of gender-related analyses. While the regression model still identified significant associations with gender, the missing data may have affected the robustness of these results. Patients in psychosomatic medicine suffer from psychological disorders, which may have influenced their preferences and behavior. Nevertheless, recent studies have shown that eHealth services are an effective and appropriate therapeutic tool [70] to reduce mental complaints [71] and stress symptoms [72].

In conclusion, the study indicates that recognition and accommodation of diverse preferences is critical in designing and implementing future eHealth services, which might be central in managed care programs. Although this investigation focused on the pandemic context, eHealth services can be expected to remain an integral component of health care delivery [73]. By integrating these findings, especially accommodating the age and gender of patients, a potential rise in patient engagement can be anticipated within the transforming eHealth environment. These results highlight the need for a tailored approach to diverse patient requirements to improve health care outcomes as eHealth services continue to progress and evolve.

## Abbreviations

VIF: variance inflation factors

## References

[1] Eysenbach G (2001). What is e-health?. J Med Internet Res.

[2] Alonso SG, Marques G, Barrachina I (2021). Telemedicine and e-Health research solutions in literature for combatting COVID-19: a systematic review. Health Technol (Berl).

[3] Puspitasari I, Firdauzy A (2019). Characterizing consumer behavior in leveraging social media for e-patient and health-related activities. Int J Environ Res Public Health.

[4] D’Anza B, Pronovost PJ (2022). Digital health: unlocking value in a post-pandemic world. Popul Health Manag.

[5] Ekeland AG, Bowes A, Flottorp S (2010). Effectiveness of telemedicine: a systematic review of reviews. Int J Med Inform.

[6] Elbert NJ, van Os-Medendorp H, van Renselaar W (2014). Effectiveness and cost-effectiveness of ehealth interventions in somatic diseases: a systematic review of systematic reviews and meta-analyses. J Med Internet Res.

[7] Furlanis G, Ajčević M, Naccarato M (2020). e-Health vs COVID-19: home patient telemonitoring to maintain TIA continuum of care. Neurol Sci.

[8] Okereafor K, Adebola O, Djehaiche R (2020). Exploring the potentials of telemedicine and other non-contact electronic health technologies in controlling the spread of the novel coronavirus disease (COVID-19). Int J IT Eng.

[9] Hollander JE, Carr BG (2020). Virtually perfect? Telemedicine for COVID-19. N Engl J Med.

[10] Chauhan V, Galwankar S, Arquilla B (2020). Novel Coronavirus (COVID-19): leveraging telemedicine to optimize care while minimizing exposures and viral transmission. J Emerg Trauma Shock.

[11] Tebeje TH, Klein J (2021). Applications of e-Health to support person-centered health care at the time of COVID-19 pandemic. Telemed J E Health.

[12] Fox S, Duggan M (2013). Health online 2013. https://www.pewresearch.org/internet/2013/01/15/health-online-2013/.

[13] Herrmann-Werner A, Weber H, Loda T (2019). “But Dr Google said…” - Training medical students how to communicate with E-patients. Med Teach.

[14] Ferguson T (2007). Patient Advocacy for Health Care Quality: Strategies for Achieving Patient-Centered Care.

[15] Forkner-Dunn J (2003). Internet-based patient self-care: the next generation of health care delivery. J Med Internet Res.

[16] Buchanan R, Beckett RD (2014). Assessment of vaccination‐related information for consumers available on Facebook. Health Info Libraries J.

[17] Grajales III FJ, Sheps S, Ho K, Novak-Lauscher H, Eysenbach G (2014). Social media: a review and tutorial of applications in medicine and health care. J Med Internet Res.

[18] Masters K (2009). Opening the non-open access medical journals: Internet-based sharing of journal articles on a medical web site. Internet J Med Inform.

[19] Masters K (2009). Opening the closed-access medical journals: Internet- based sharing of institutions’ access codes on a medical website. Internet J Med Inform.

[20] Swab M, Romme K (2016). Scholarly sharing via Twitter: #icanhazpdf requests for health sciences literature. J Can Health Libr Assoc.

[21] Channa A, Popescu N, Skibinska J, Burget R (2021). The rise of wearable devices during the COVID-19 pandemic: a systematic review. Sensors (Basel).

[22] Masters K (2017). Preparing medical students for the e-patient. Med Teach.

[23] Nanda M, Sharma R (2021). A review of patient satisfaction and experience with telemedicine: a virtual solution during and beyond COVID-19 pandemic. Telemed J E Health.

[24] Ndwabe H, Basu A, Mohammed J (2024). Post pandemic analysis on comprehensive utilization of telehealth and telemedicine. Clinical eHealth.

[25] Alsabeeha NHM, Atieh MA, Balakrishnan MS (2023). Older adults’ satisfaction with telemedicine during the COVID-19 pandemic: a systematic review. Telemed J E Health.

[26] Pappot N, Taarnhøj GA, Pappot H (2020). Telemedicine and e-Health solutions for COVID-19: patients’ perspective. Telemed J E Health.

[27] World Medical Association (2013). World Medical Association Declaration of Helsinki: ethical principles for medical research involving human subjects. JAMA.

[28] Masters K, Loda T, Al-Abri R, Johannink J, Herrmann-Werner A (2020). Surgical patients’ use of, and attitudes towards, the internet for e-patient activities in Germany and Oman. Ann Med Surg (Lond).

[29] Masters K, Loda T, Johannink J, Al-Abri R, Herrmann-Werner A (2020). Surgeons’ interactions with and attitudes toward e-patients: questionnaire study in Germany and Oman. J Med Internet Res.

[30] Fonteyn ME, Kuipers B, Grobe SJ (1993). A description of think aloud method and protocol analysis. Qual Health Res.

[31] (2021). IBM SPSS statistics for windows, version 280. IBM Corp.

[32] Chandrasekaran R (2024). Telemedicine in the post-pandemic period: understanding patterns of use and the influence of socioeconomic demographics, health status, and social determinants. Telemed J E Health.

[33] Monaghesh E, Hajizadeh A (2020). The role of telehealth during COVID-19 outbreak: a systematic review based on current evidence. BMC Public Health.

[34] Mills EC, Savage E, Lieder J, Chiu ES (2020). Telemedicine and the COVID-19 pandemic: are we ready to go live?. Adv Skin Wound Care.

[35] Selvam KP, Kosalram K, Chinnaiyan S (2024). Post-COVID pandemic: the new normal and aftermath. J Family Med Prim Care.

[36] Snoswell CL, Smith AC, Page M, Caffery LJ (2023). Patient preferences for specialist outpatient video consultations: a discrete choice experiment. J Telemed Telecare.

[37] von Weinrich P, Kong Q, Liu Y (2024). Would you Zoom with your doctor? A discrete choice experiment to identify patient preferences for video and in-clinic consultations in German primary care. J Telemed Telecare.

[38] Iyengar K, Mabrouk A, Jain VK, Venkatesan A, Vaishya R (2020). Learning opportunities from COVID-19 and future effects on health care system. Diabetes Metab Syndr Clin Res Rev.

[39] Matusitz J, Breen GM (2007). Telemedicine: its effects on health communication. Health Commun.

[40] Ahmed Kamal M, Ismail Z, Shehata IM (2023). Telemedicine, E-Health, and multi-agent systems for chronic pain management. Clin Pract.

[41] Tang M, Reddy A (2022). Telemedicine and its past, present, and future roles in providing palliative care to advanced cancer patients. Cancers (Basel).

[42] Holida ND, Nugroho SA (2022). Telemedicine in palliative care during the COVID-19 era: a review of the literature. JHNS.

[43] Coleman C (2020). Health literacy and clear communication best practices for telemedicine. HLRP Health Lit Res Pract.

[44] (2022). Consolidated Telemedicine Implementation Guide.

[45] Zhao Y, Zhang J (2017). Consumer health information seeking in social media: a literature review. Health Info Libraries J.

[46] Luo A, Qin L, Yuan Y (2022). The effect of online health information seeking on physician-patient relationships: systematic review. J Med Internet Res.

[47] Tan SSL, Goonawardene N (2017). Internet health information seeking and the patient-physician relationship: a systematic review. J Med Internet Res.

[48] O’Donnell RR (2010). Stepped Care and E-Health: Practical Applications to Behavioral Disorders.

[49] Puspitasari I The impacts of consumer’s health topic familiarity in seeking health information online.

[50] Starcevic V, Berle D (2013). Cyberchondria: towards a better understanding of excessive health-related Internet use. Expert Rev Neurother.

[51] Montgomery L (2015). Supporting radiation therapy patients with limited health literacy. J Med Imaging Radiat Sci.

[52] Mackert M, Mabry-Flynn A, Champlin S, Donovan EE, Pounders K (2016). Health literacy and health information technology adoption: the potential for a new digital divide. J Med Internet Res.

[53] Huang Y, Yang C (2020). A metacognitive approach to reconsidering risk perceptions and uncertainty: understand information seeking during COVID-19. Sci Commun.

[54] Alyafei K, Ahmed R, Abir FF, Chowdhury MEH, Naji KK (2022). A comprehensive review of COVID-19 detection techniques: from laboratory systems to wearable devices. Comput Biol Med.

[55] Panicker RM, Chandrasekaran B (2022). “Wearables on vogue”: a scoping review on wearables on physical activity and sedentary behavior during COVID-19 pandemic. Sport Sci Health.

[56] Khondakar KR, Kaushik A (2022). Role of wearable sensing technology to manage long COVID. Biosensors (Basel).

[57] Kang HS, Exworthy M (2022). Wearing the future-wearables to empower users to take greater responsibility for their health and care: scoping review. JMIR Mhealth Uhealth.

[58] Ratheesh A, Alvarez-Jimenez M (2022). The Future of Digital Mental Health in the Post-Pandemic World: Evidence-Based, Blended, Responsive and Implementable.

[59] Huhn S, Axt M, Gunga HC (2022). The impact of wearable technologies in health research: scoping review. JMIR Mhealth Uhealth.

[60] Fittler A, Ambrus T, Serefko A (2022). Attitudes and behaviors regarding online pharmacies in the aftermath of COVID-19 pandemic: at the tipping point towards the new normal. Front Pharmacol.

[61] Long CS, Kumaran H, Goh KW (2022). Online pharmacies selling prescription drugs: systematic review. Pharmacy (Basel).

[62] Aronson JK, Ferner RE, Richards GC (2022). Deaths attributed to the use of medications purchased online. BMJ Evid Based Med.

[63] Ahmed J, Modica de Mohac L, Mackey TK, Raimi-Abraham BT (2022). A critical review on the availability of substandard and falsified medicines online: incidence, challenges and perspectives. J Med Access.

[64] Pogorzelska K, Chlabicz S (2022). Patient satisfaction with telemedicine during the COVID-19 pandemic-a systematic review. Int J Environ Res Public Health.

[65] Herrler A, Kukla H, Vennedey V, Stock S (2022). Which features of ambulatory healthcare are preferred by people aged 80 and over? Findings from a systematic review of qualitative studies and appraisal of confidence using GRADE-CERQual. BMC Geriatr.

[66] Albarrak A, Alhaidar F, Alanazi M, Bamarei N, Alarifi J, Obaid NB (2024). Satisfaction and literacy of telepsychiatry among adult patients attending psychiatry outpatient clinics in an academic hospital. J Nat Sci Med.

[67] Zein SH, Zein M, Ilmi B, Febriana SKT, Suhartono E, Rofii A (2022). Meta-analysis: relationship of age, sex, time of serving, and consultation modalitas with patient satisfaction on telemedicine use. Int j health med sci.

[68] Carrieri V, De Paola M, Gioia F (2021). The health-economy trade-off during the COVID-19 pandemic: communication matters. PLoS ONE.

[69] Lee MK, Basford JR, Heinemann AW, Cheville A (2020). Assessing whether ad hoc clinician-generated patient questionnaires provide psychometrically valid information. Health Qual Life Outcomes.

[70] Postel MG, de Haan HA, De Jong CAJ (2008). E-therapy for mental health problems: a systematic review. Telemed J E Health.

[71] Christensen H, Hickie IB (2010). Using e-health applications to deliver new mental health services. Med J Aust.

[72] Stratton E, Lampit A, Choi I, Calvo RA, Harvey SB, Glozier N (2017). Effectiveness of eHealth interventions for reducing mental health conditions in employees: a systematic review and meta-analysis. PLoS ONE.

[73] Chen A, Ayub MH, Mishuris RG (2023). Telehealth policy, practice, and education: a position statement of the Society of General Internal Medicine. J GEN INTERN MED.

